# Long non-coding RNA BC002811 Promotes Gastric Cancer Metastasis by Regulating SOX2 Binding to the *PTEN* Promoter

**DOI:** 10.7150/ijbs.76407

**Published:** 2023-01-16

**Authors:** Xiaocong Lin, Guodan Li, Xiuwen Yan, Weiyu Fu, Jie Ruan, Hang Ding, Huajun Yu, Xiaoyi Chen, Liubo Lan, Yong Dai, Kai Pan, Xinguang Liu, Haitao Zhang

**Affiliations:** 1 Guangdong Provincial Key Laboratory of Medical Molecular Diagnostics, Institute of Biochemistry and Molecular Biology, Guangdong Medical University, Zhanjiang 524023, Guangdong, China.; 2 Peptide and Protein Research and Application Key Laboratory of Guangdong Medical University, Zhanjiang 524023, Guangdong, China.; 3 Clinical Medical Research Center, Shenzhen People's Hospital, Shenzhen 518020, Guangdong, China.; 4 Department of Gastrointestinal Surgery, Shenzhen People's Hospital, Shenzhen 518020, Guangdong, China.

**Keywords:** Long non-coding RNA, Gastric cancer, Tumor metastasis, PTEN

## Abstract

There is increasing evidence that long non-coding RNAs (lncRNAs) are involved in the pathogenesis and progression of gastric cancer (GC), however, the underlying mechanisms remain poorly understood. In this study, we identified lncRNA BC002811 as a critical regulator of GC development and progression. BC002811 was upregulated in GC tissues and cell lines, and that high expression of BC002811 was indicative of a reduction in overall survival of GC patients. Our research reveals that BC002811 promoted GC cell proliferation, migration, invasion, and inhibition of apoptosis *in vitro*, as well as accelerated tumor growth and metastasis *in vivo*. We also found that BC002811 upregulated MMP2 and MMP9 and promoted GC cell metastasis partially through downregulating PTEN expression. BC002811 may act as a molecular decoy for the transcription factor SOX2, thereby inhibiting the transcription of PTEN by blocking SOX2 binding to the *PTEN* promoter. Our study advances the understanding of the role of BC002811 in the pathogenesis of GC and provides new molecular targets for therapeutic intervention against GC metastasis.

## Introduction

Gastric cancer (GC) is one of the most common human malignancies worldwide, with more than 950,000 new cases are diagnosed every year, particularly in East Asia and Eastern Europe 1,2. Despite several advances in chemotherapy, radiotherapy, and surgical techniques for GC over the past decades, 40% of GC patients develop metastases, and only just over 5% of these patients survive for more than 5 years 1,3. Therefore, further investigation of the molecular pathogenesis of GC metastasis, with the aim of developing new therapeutic strategies, is urgently needed.

Long non-coding RNAs (lncRNAs) are endogenous transcripts longer than 200 nucleotides that lack protein-coding capabilities 2. However, studies indicate that most lncRNAs have short open reading frames, which gives them the potential to encode functional micropeptides 4. LncRNAs are key regulators of gene expression, participating in a range of cellular and physiological processes, such as dosage compensation, genomic imprinting, chromatin packaging, mRNA processing, cell differentiation, and embryonic development 5. LncRNAs regulate cellular processes involved in genetic mutations, alternative splicing, mRNA stability, miRNA sponging, transcription factor interactions, and epigenetic modification 6,7. There is increasing evidence that aberrant lncRNAs play central roles in promoting proliferation, apoptosis, invasion, metastasis, angiogenesis, and drug resistance in various types of cancers 8,9. Thus, lncRNAs have potential as new biomarkers and pharmaceutical targets in oncology.

By comparing tumors to matched adjacent non-tumorous tissues using lncRNA microarrays, we previously demonstrated that a number of lncRNAs are abnormally expressed in GC 10. Among these differentially expressed lncRNAs, lncRNA BC002811 was found to be highly expressed in GC tissues. BC002811 has been reported to be downregulated in astrocytoma tissues, and lower BC002811 levels are significantly associated with poor patient prognosis 11. However, the function and mechanism of BC002811 in gastric cancer need to be further studied.

In this study, we found that BC002811 expression was upregulated in GC tissues and cell lines. High BC002811 expression correlated with poor prognosis in patients with GC. By altering the expression of BC002811 in GC cells, we demonstrated that BC002811 increased proliferation, reduced apoptosis and promoted metastasis. Specifically, we propose that the pro-metastatic behavior of BC002811 may be through inhibition of the transcription of PTEN, through disruption of the interplay between the transcription factor SOX2 and the *PTEN* promoter.

## Materials and Methods

### Tissue microarrays (TMA) and *in situ* hybridization (ISH)

Human GC and corresponding non-tumorous tissue microarrays (TMA) were purchased from Shanghai Outdo Biotech (Shanghai, China). *In situ* hybridization (ISH) was performed using a digoxigenin (DIG)-labeled lncRNA BC002811 probe (Exiqon, Vedbaek, Denmark) following the manufacturer's instructions. Deparaffinized, graded alcohol-treated tissue sections were digested with proteinase K (40 μg/ml) at 37°C for 20 min, fixed with paraformaldehyde (4%; 10 min), incubated with prehybridization solution (53°C; 2 h), and then hybridized with the DIG-labeled probe for 24 h. After incubating with blocking solution (25°C; 1 h), sections were incubated overnight (4°C) with an alkaline phosphatase (AP)-conjugated anti-DIG antibody (Roche, Basel, Switzerland) and stained with AP substrate NBT/BCIP (Pierce, Rockford, IL, USA) for 4 h followed by nuclear fast red counterstaining. Image J software was used for quantitative analysis of the hybridization signals of the GC TMAs. According to the description of Chen et al. 12, a staining score was obtained by multiplying the extent (0-100) and intensity (0-3) of positive staining for each sample point. All sections were evaluated by two blinded pathologists.

### Tumor xenografts and lung metastasis in nude mice

The function of BC002811 in tumorigenesis and metastasis *in vivo* was investigated using 4-6 weeks-old male BALB/c nude mice (Vital River Laboratory Animal Technology Co. Ltd., Beijing, China). The cell lines used for the *in vivo* experiments were labeled with luciferase. For the tumor formation assay, the left or right thigh of each mouse was injected with 5 × 10^6^ cells. All tumor xenografts were weighed and fixed for immunohistochemical analysis. For the lung metastasis model, mice were injected with 2 × 10^6^ cells through the tail vein. After intraperitoneal injected of 150 mg/kg of D-luciferin, each mouse was imaged using an IVIS^®^ Lumina II imaging system (Caliper Life Sciences, Hopkinton, MA, USA). All animal experiments were approved by the Laboratory Animal Ethics Committee of Guangdong Medical University.

### Immunohistochemistry (IHC)

GC tissue sections were incubated with hydrogen peroxide (3%) for 10 min, heated to 100°C in citrate buffer for 8 min for antigen retrieval, and subsequently cooled to 25°C. Sections were incubated with goat serum (10%; 30min) in a humidified environment to block non-specific binding. Subsequently, the sections were incubated overnight (4°C) with primary antibodies, incubated with secondary antibodies (25°C; 30 min) in a humid chamber, developed with diaminobenzidine tetrahydrochloride (DAB), stained with hematoxylin, and imaged. The immunostaining of GC TMAs was quantified by multiplying the staining intensity by the percentage of area with positive staining 12. All samples were evaluated by two blinded pathologists.

### qPCR array

The qPCR for microarray assay was carried out using the RT^2^ Profiler PCR Array [Human Tumor Metastasis (PAHS-028Z)] following the manufacturer's protocols (Qiagen, Hilden, Germany). The Human Tumor Metastasis qPCR Array allows simultaneous analysis of expression profiling of 84 key genes known to be related to human tumor metastasis. For signal detection, an initial denaturetion step of 10 min at 95°C was followed by 40 cycles each at 95°C for 15 s and 60°C for 1 min using the ABI Prism^®^ 7900HT Real-Time PCR System (Applied Biosystems, CA, USA). The RNA levels were normalized to five housekeeping genes. Fold changes in mRNA abundance were calculated according to the 2^-∆∆Ct^ method 13, with a fold-change threshold set at 2.0.

### RNA pull-down

The RNA pull-down protocol was as previously described 14. Biotin-labeled sense and antisense RNA of BC002811 transcribed *in vitro* with T7 RNA polymerase (Roche, Basel, Switzerland) and Biotin RNA Labeling Mix (Roche) were incubated with cell protein extracts to allow the formation of an RNA-protein binding mixture, which was then captured on streptavidin beads. After SDS-PAGE and silver staining of the washed beads, proteins differentially bound to RNA were identified by mass spectrometry, and selected specific proteins were detected by western blotting.

### RNA immunoprecipitation (RIP)

RIP was carried out using a Magna RIP™ RNA-Binding Protein Immunoprecipitation Kit (Millipore, Bedford, MA, USA) following the manufacturer's protocol. Protein A/G magnetic beads resuspended in lysis buffer were coupled to anti-SOX2 antibody or normal rabbit IgG (negative control) by incubating at 4°C for 4 h. The magnetic beads with the coupled antibodies were then incubated with the lysis mixture (4°C; 6 h), and the RNA was eluted, and RT-PCR was performed. The detection primers for BC002811 are shown in Table S1.

### RNA-fluorescent *in situ* hybridization (RNA-FISH) combined with immunofluorescence analysis

GC cells that had reached 60%-70% confluence on coverslips in 12-well plates were fixed with paraformaldehyde (4%) for 15 min, followed by quenching of autofluorescence three times with 0.1 M glycine for 10 min each, permeabilized with Triton X-100 (0.4%; 15min), treated with acetic anhydride (0.25%) for 10 min, and hybridized with Cy3-labeled oligonucleotide probes in hybridization solution at 45°C for 16 h. GC cells were then blocked with BSA (1%; 37°C) for 1 h, incubated overnight (4 °C) with anti-SOX2 antibody (1:200, Abcam, Cambridge, UK), and with Cy3-labeled secondary antibody (2 h; 25°C). The nuclei were stained with 4,6-diamidino-2-phenylindole (DAPI) and the cells were imaged using a TCS SPE confocal laser-scanning microscope (Leica, Mannheim, Germany).

### Chromatin Immunoprecipitation (ChIP)

The ChIP assay was carried out using the EZ ChIP™ Chromatin Immunoprecipitation Kit (Millipore) following the manufacturer's instructions. The cells were cross-linked with formaldehyde (1%) and quenched with 125 mM glycine. The cross-linked chromatin was then sheared into 200-1,000bp fragments and precipitated with antibodies against SOX2. Fragmented chromatin DNA (10%) was used as the input, and normal rabbit IgG was used as a negative control. Fold enrichment of DNA was determined by qPCR with PTEN promoter-specific primers (Table S1).

### Electrophoretic mobility shift assay (EMSA)

EMSA was conducted using a LightShift™ Chemiluminescent EMSA Kit (Pierce, Rockford, IL, USA). Nuclear proteins were extracted using NE-PER Nuclear and Cytoplasmic Extraction Reagent (Pierce, Rockford, IL, USA). The nuclear proteins in the binding buffer were then incubated with or without excess unlabeled wild-type competitors, followed by the addition of the biotin-labeled probes. For the supershift reaction, before the addition of the probes, an antibody against SOX2 was added to the reaction mixture. All protein-DNA complexes were separated by electrophoresis on a non-denaturing polyacrylamide gel and transferred to a membrane. Finally, antigens bound to the membranes were visualized by chemiluminescent detection.

### Supplementary Material

Details regarding additional materials and methods used in this study are provided in the Supplementary Material.

## Results

### Upregulation of BC002811 expression in GC and significant correlation with poor survival

**Figure 1:**
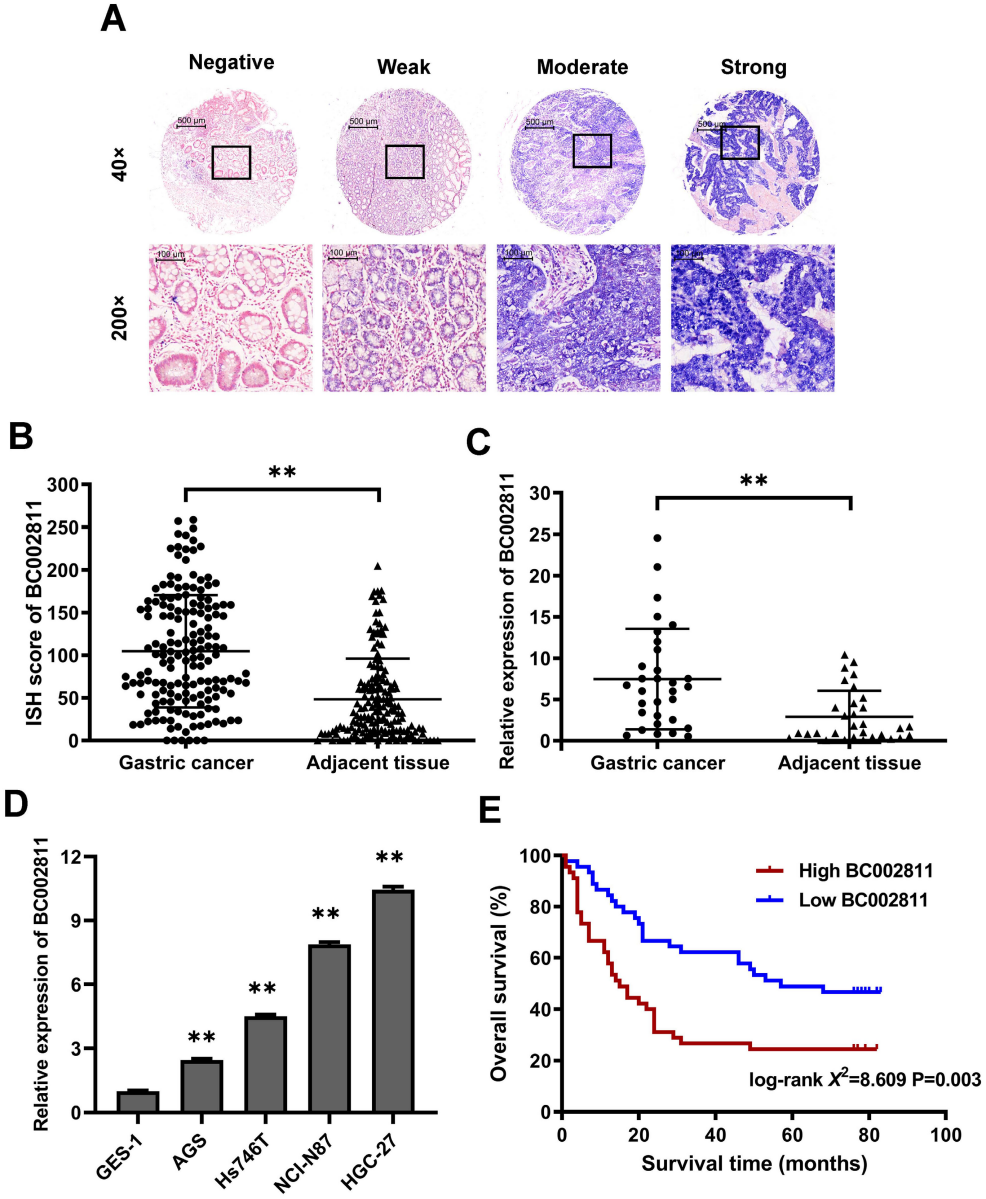
** Elevated expression of lncRNA BC002811 in GC. (A)** Representative images of different intensities of BC002811 ISH staining in tissue microarrays (40× and 200× magnification). Scale bars, 500 μm (40× images) and 100 μm (200× images). **(B)** ISH scores for BC002811 staining in GC tissue microarrays. **(C-D)** Expression of BC002811 was analyzed by qPCR in GC tissues **(C)** and GC cells **(D)**. **(E)** Kaplan-Meier survival analysis of overall survival in GC patients according to BC002811 expression. All GC patients were segregated into “High BC002811” or “Low BC002811” expression groups using the median expression level of BC002811 as the cut-off point. Data expressed as mean±SD. **P <0.01.

In our previous study, we compared GC tumor samples to matched adjacent non-tumorous samples using lncRNA microarrays, and found that BC002811 expression was upregulated in GC 10. To confirm this increase, the expression levels of BC002811 were evaluated by *in situ* hybridization (ISH) in 163 pairs of clinical GC samples and adjacent non-tumorous gastric tissues using tissue microarrays (Fig. S1A-B). BC002811 staining intensity was classified as negative, weak, moderate, or strong (Fig. 1A). The BC002811 ISH score was higher in GC tissues compared with adjacent noncancerous tissues (Fig. 1B). Moreover, an association between high BC002811 levels and tumor size, TNM stage, and lymph node metastasis was found (Table S2). To verify our findings, we examined BC002811 expression by qPCR in a panel of 31 paired surgically resected human GC/peritumoral samples along with four GC cell lines and the normal gastric epithelial cell line GES-1. The expression of BC002811 was consistently higher in GC tissues compared to the corresponding adjacent tissues (Fig. 1C). We also found that there was increased expression of BC002811 in AGS, Hs746T, HGC-27, and NCI-N87 cells compared with GES-1 cells (Fig. 1D). To explore whether BC002811 could be a crucial factor in determining the clinical outcomes of GC patients, Kaplan-Meier analysis and log-rank testing were utilized to assess the impact of BC002811 expression on the overall survival (OS) of GC patients. The OS of GC patients with high BC002811 expression (median OS: 15 months) was found to be substantially shorter than the OS of those with low BC002811 expression (median OS: 57 months; *P* = 0.003; Fig. 1E).

### BC002811 exerts a significant effect on GC cell proliferation, apoptosis, migration, and invasion *in vitro*

**Figure 2:**
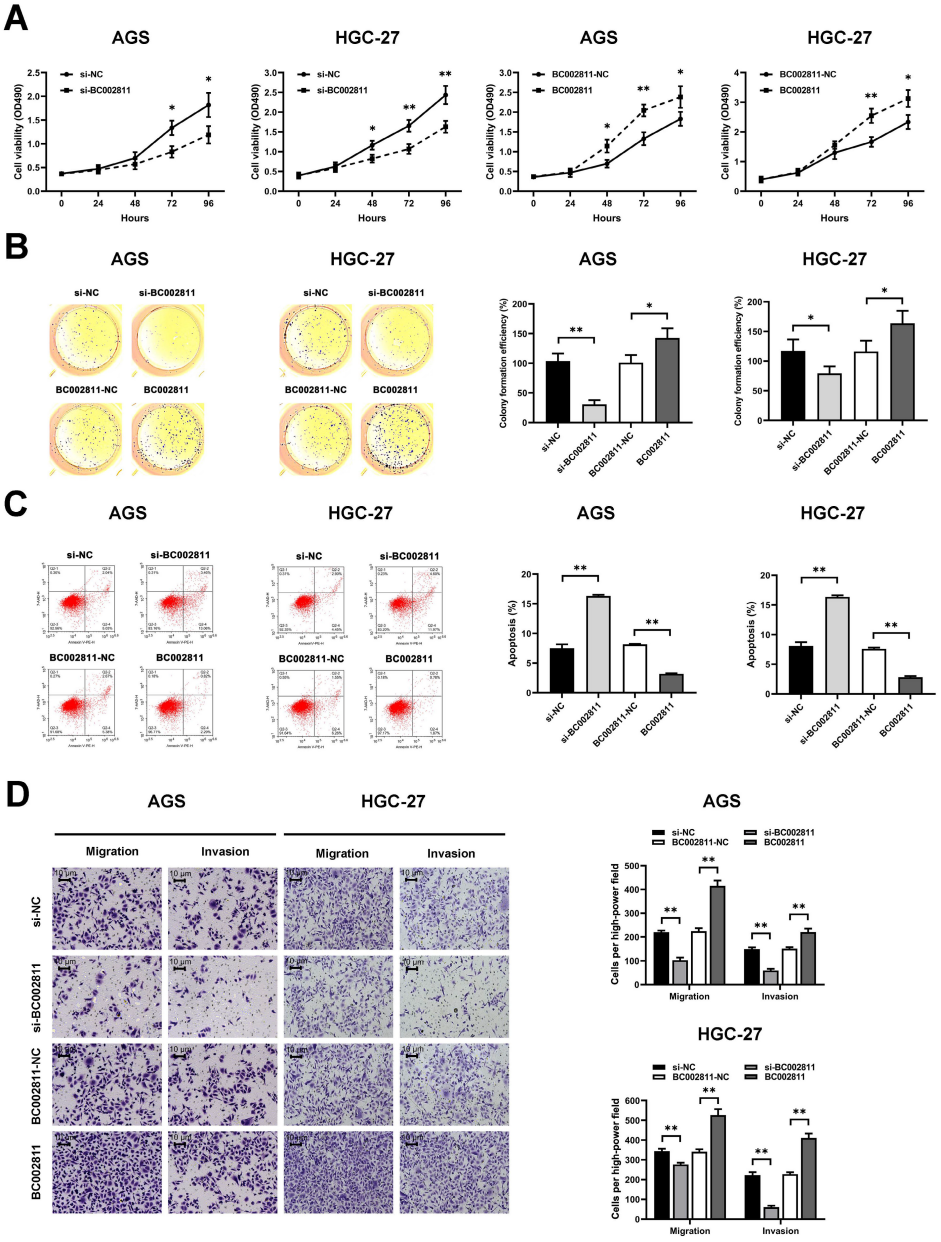
** BC002811 promotes cell proliferation, migration, and invasion, and inhibits cell apoptosis in GC cells.** AGS and HGC-27 cells were transfected with siRNA against BC002811 (si-BC002811) or scramble-control siRNA (si-NC), or BC002811 expression vector (BC002811) or the empty vector (BC002811-NC).** (A)** MTS assays were performed to determine the viability of AGS and HGC-27 cells. **(B)** Colony formation assays were performed to determine the colony formation efficiencies of AGS and HGC-27 cells. **(C)** Flow cytometric analysis of the effect of BC002811 on AGS and HGC-27 cell apoptosis. **(D)** Transwell^®^ assays showed that knockdown of BC002811 inhibited migration and invasion of AGS and HGC-27 cells, while ectopic expression of BC002811 resulted in the opposite effects. Scale bar, 10 μm. Data expressed as mean±SD. *P < 0.05, **P <0.01.

To evaluate the effects of BC002811 on the key biological properties of GC cells, AGS and HGC-27 cells transfected with a BC002811 overexpression vector or small interfering RNAs (siRNAs) targeting BC002811 were analyzed. MTS assays showed that cell proliferation was diminished in AGS and HGC-27 cells in which BC002811 had been knocked down (Fig. 2A). In keeping with reduced cell proliferation, AGS and HGC-27 cells in which BC002811 had been silenced also exhibited considerably decreased colony formation efficiency compared with control cells (Fig. 2B). By contrast, exogenous expression of BC002811 increased proliferation and colony formation in AGS and HGC-27 cells (Fig. 2A-B). Flow cytometry showed that downregulation of BC002811 induced apoptosis in AGS and HGC-27 cells, whereas upregulation of BC002811 expression decreased apoptosis (Fig. 2C). We then used the Transwell^®^ assay to investigate the effect of BC002811 on cell invasion and migration. BC002811 overexpression led to a pronounced increase in migrating and invading cell counts, while knockdown of BC002811 produced the opposite results (Fig. 2D). The above findings indicate that BC002811 expression is tightly correlated with GC cell proliferation, apoptosis, invasion, and migration *in vitro*.

### BC002811 promotes tumorigenesis and metastasis of GC cells *in vivo*

**Figure 3:**
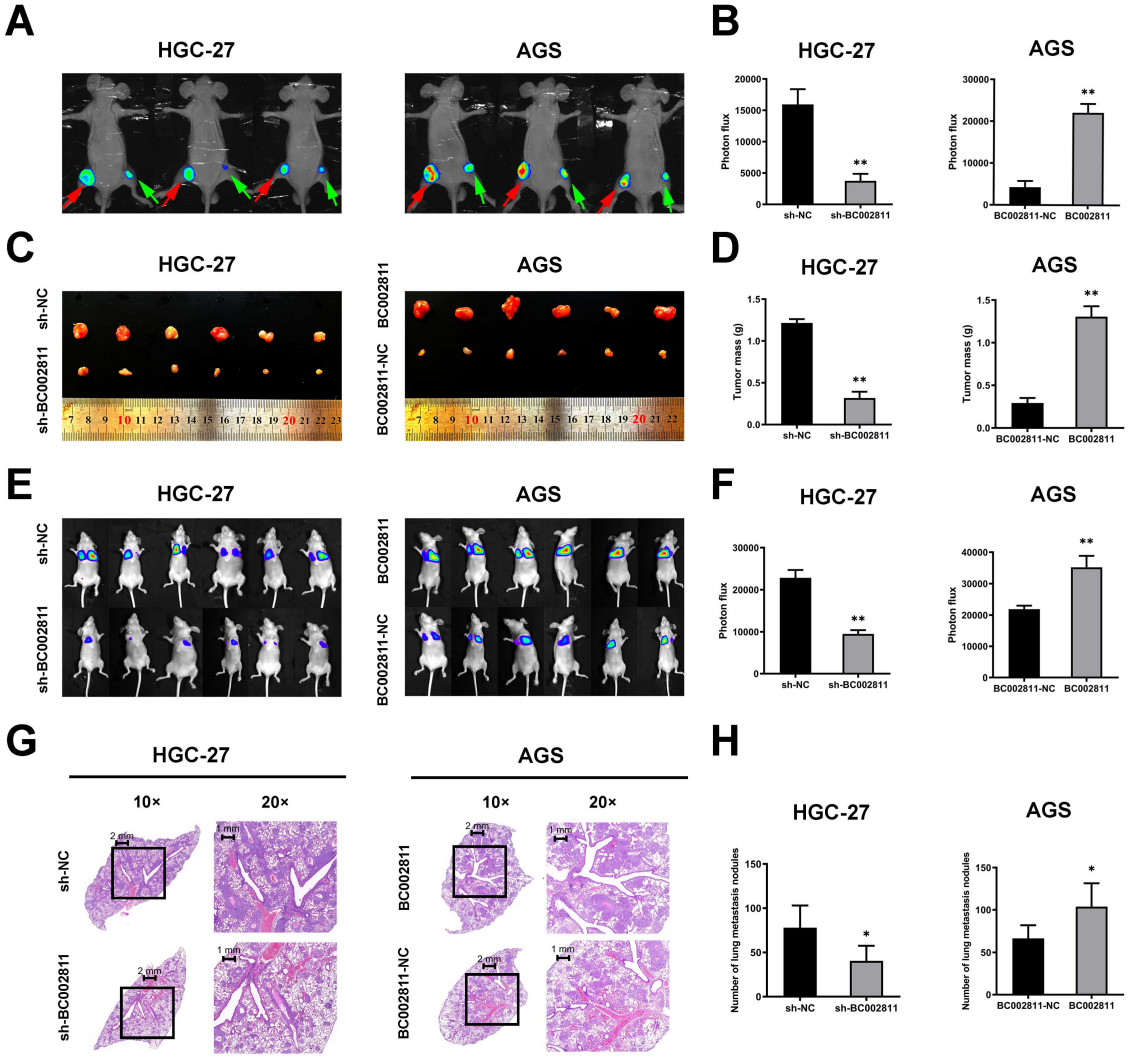
** BC002811 regulates tumor growth and metastasis *in vivo*. (A)** Representative images of tumor xenografts formed in nude mice following injection of HGC-27 and AGS cells viewed with an IVIS^®^ Lumina II Imaging System. The luciferase signals were imaged in mice bearing tumors from BC002811 stable knockdown (green arrow) or control (red arrow) HGC-27 cells, and BC002811 stable overexpressing (red arrow) or control (green arrow) AGS cells. **(B)** Statistical analysis of the luciferase signals in mouse xenograft models. **(C)** Images and **(D)** weights of tumor xenografts from nude mice in each group. **(E)** Images of mice in each group after tail vein injections of HGC-27 or AGS cells. **(F)** The luciferase signals of mice in each group after tail vein injections. **(G)** Hematoxylin and eosin-stained (H&E) images of lungs harvested from nude mice in each treatment group. Scale bars, 2 mm (10× images) and 1 mm (20× images).** (H)** Number of lung metastasis nodules. Data expressed as mean±SD. *P < 0.05, **P <0.01.

To assess the effects of BC002811 on tumorigenesis and metastasis of GC *in vivo*, HGC-27 cells with stable BC002811 knockdown, AGS cells stably overexpressing BC002811, and the respective control cells were injected into nude mice. As shown in Fig. 3A-C, mice injected with cells that had stable BC002811 knockdown displayed noticeable inhibition of tumor xenograft growth compared with mice injected with control cells. The tumor weight in the sh-BC002811 group was also significantly lower compared to the control group (Fig. 3D). Conversely, stable overexpression of BC002811 increased tumor growth (Fig. 3A-D). Likewise, the results of the tail vein metastasis assay *in vivo* showed that the animals in the BC002811 stable knockdown group exhibited fewer lung metastases, while the opposite effect was observed in the BC002811 stable overexpression group, which was confirmed by H&E staining of lung tissue slices (Fig. 3E-H). These data indicate that BC002811 increases oncogenic activity and has metastasis-promoting effects *in vivo*.

### MMP2, MMP9, and PTEN are key downstream targets of BC002811 involved in GC metastasis

**Figure 4:**
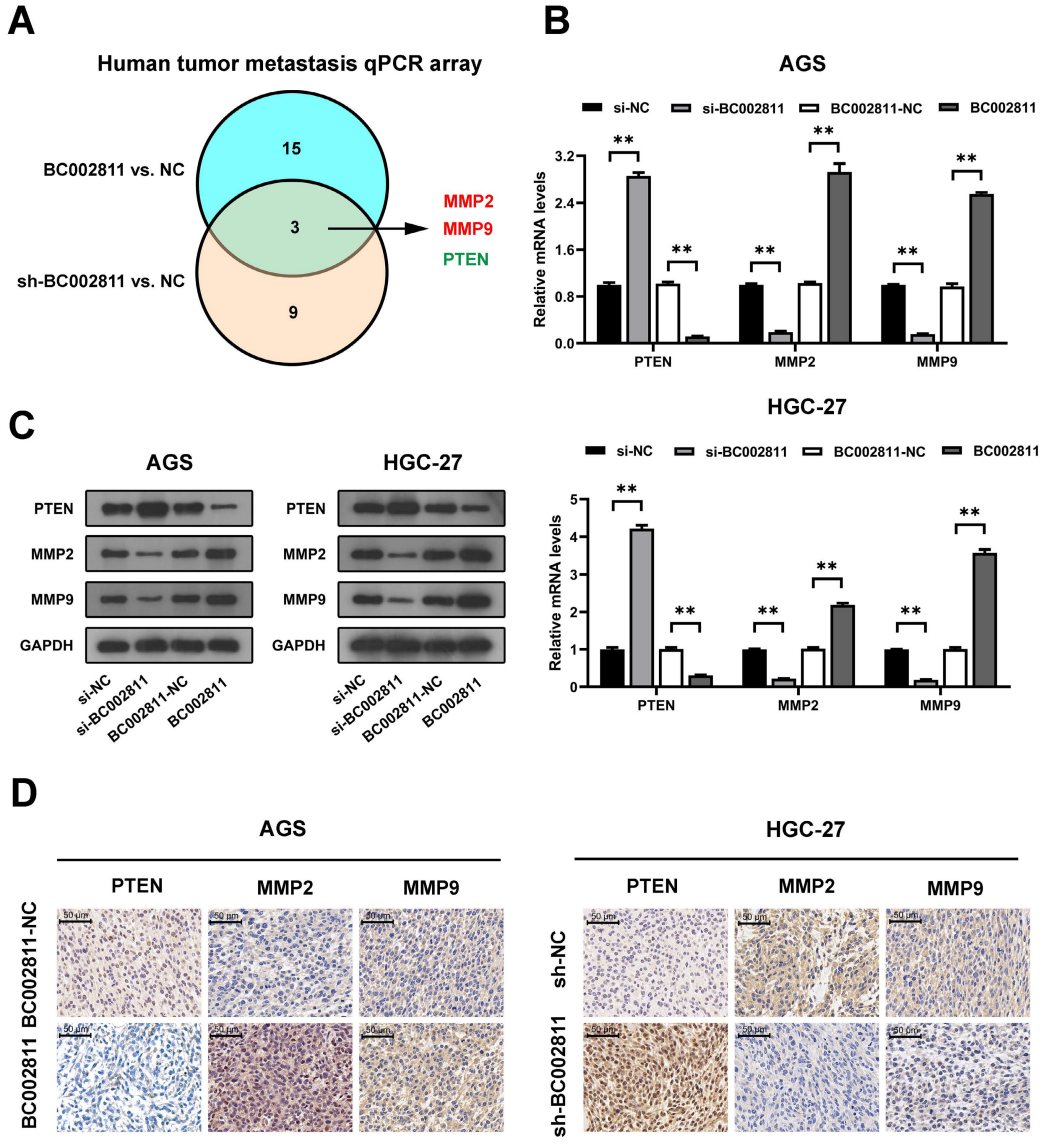
** PTEN, MMP2, and MMP9 are downstream targets of BC002811. (A)** Venn diagram of overlapping genes obtained from differentially expressed mRNAs displaying the opposite trend between the BC002811-overexpression group and the BC002811-shRNA group in human tumor metastasis qPCR arrays assay. **(B)** qPCR analysis of PTEN, MMP2, and MMP9 expression in AGS and HGC-27 cells after knockdown or overexpression of BC002811. **(C)** PTEN, MMP2, and MMP9 protein levels in AGS and HGC-27 cells by western blot after knockdown or overexpression of BC002811. **(D)** Representative IHC staining images of PTEN, MMP2, and MMP9 in subcutaneous tumor xenograft tissues. Scale bar, 100 μm. Data expressed as mean±SD. **P <0.01.

To further investigate the genes that may be involved in the promotion of metastasis by BC002811 in GC, we used human tumor metastasis qPCR arrays. As shown in Fig. 4A and Table S3, BC002811 regulated three differentially expressed genes (*MMP2*, *MMP9*, and *PTEN*) out of 84 key genes involved in human tumor metastasis. MMP2 and MMP9 are well-known tumor metastasis markers, while *PTEN* is a well-characterized tumor suppressor gene that plays a major role in the metastasis of GC 15-17. Therefore, MMP2, MMP9, and PTEN were selected for further investigation. qPCR showed that overexpression of BC002811 in AGS and HGC-27 cells led to a significant reduction of PTEN mRNA expression and a remarkable increase in MMP2 and MMP9 mRNA expression, while knockdown of BC002811 had the opposite effects (Fig. 4B). The regulation of these genes by BC002811 was further corroborated by western blot (Fig. 4C) and immunohistochemical (IHC) assays (Fig. 4D). These results hence confirm our earlier finding that BC002811 promotes GC metastasis.

### BC002811 regulates GC cell migration and invasion in part by controlling PTEN expression

**Figure 5:**
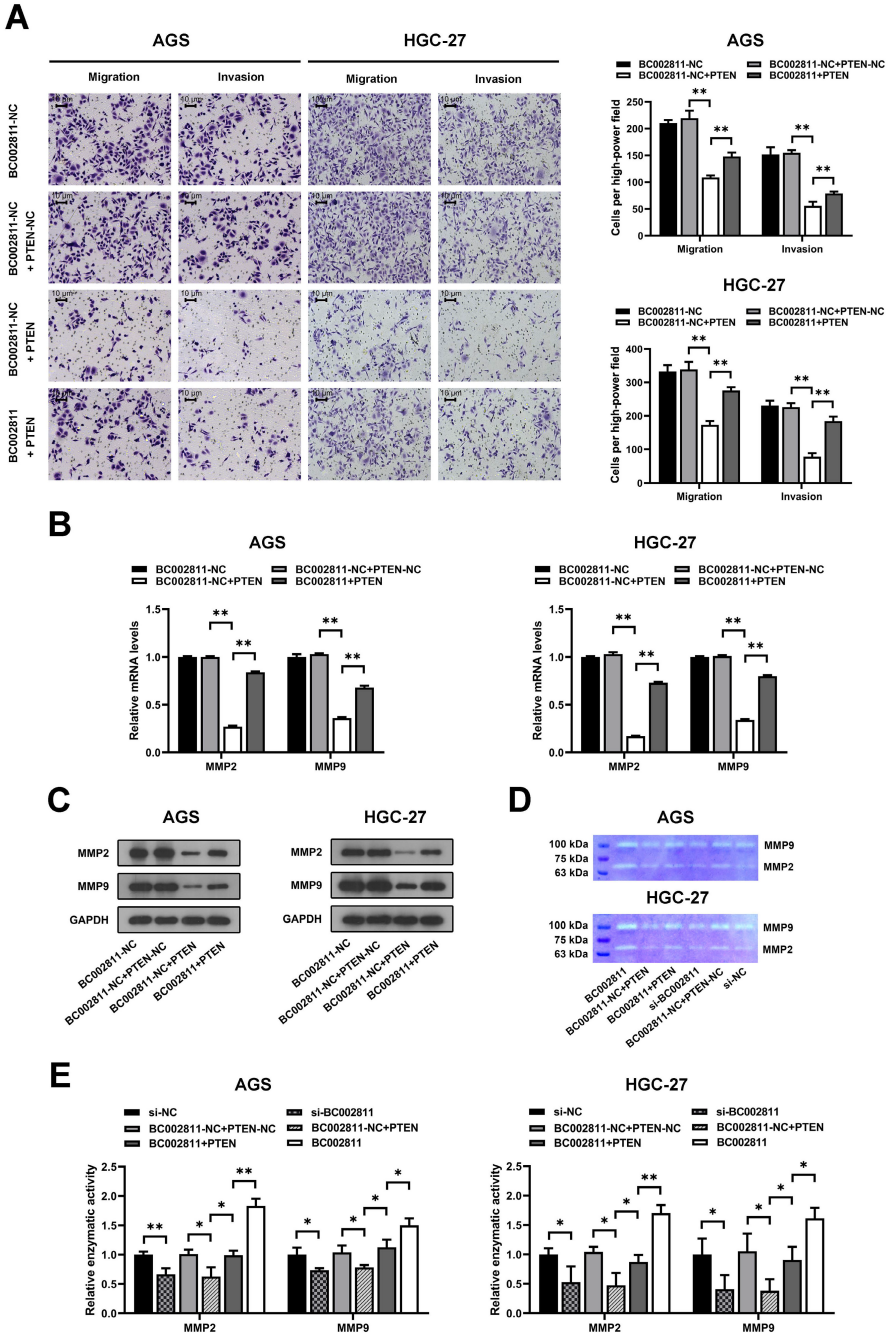
** BC002811 promotes GC cell migration and invasion in part by regulating PTEN. (A)** Transwell^®^ migration and invasion assays were carried out in PTEN-upregulated AGS and HGC-27 cells with overexpression of BC002811. Scale bar, 10 μm. qPCR **(B)** and western blot **(C)** analyses were performed to determine MMP2 and MMP9 expression after upregulation of BC002811 in PTEN-overexpressing AGS and HGC-27 cells. **(D)** Gelatin zymography was used to determine the MMP2 and MMP9 enzymatic activity in AGS and HGC-27 cells. **(E)** Statistical analysis of the gelatin zymography results. Data expressed as mean±SD. *P < 0.05, **P <0.01.

To explore whether PTEN is involved in the metastasis-promoting function of BC002811, PTEN was overexpressed in the AGS and HGC-27 GC cell lines. Transwell^®^ assays revealed that upregulation of PTEN suppressed the invasion and migration of AGS and HGC-27 cells, and that BC002811 overexpression reversed this effect (Fig. 5A). In keeping with this, BC002811 overexpression also abolished the PTEN-mediated inhibition of MMP2/MMP9 expression as well as enzymatic activity of MMP2/MMP9 (Fig. 5B-E). Since PTEN is thought to have a critical function in the regulation of GC metastasis 15-17, our findings indicate that BC002811 acts, at least in part, via PTEN to regulate metastasis in GC.

### BC002811 suppresses the expression of PTEN by interacting with SOX2

**Figure 6:**
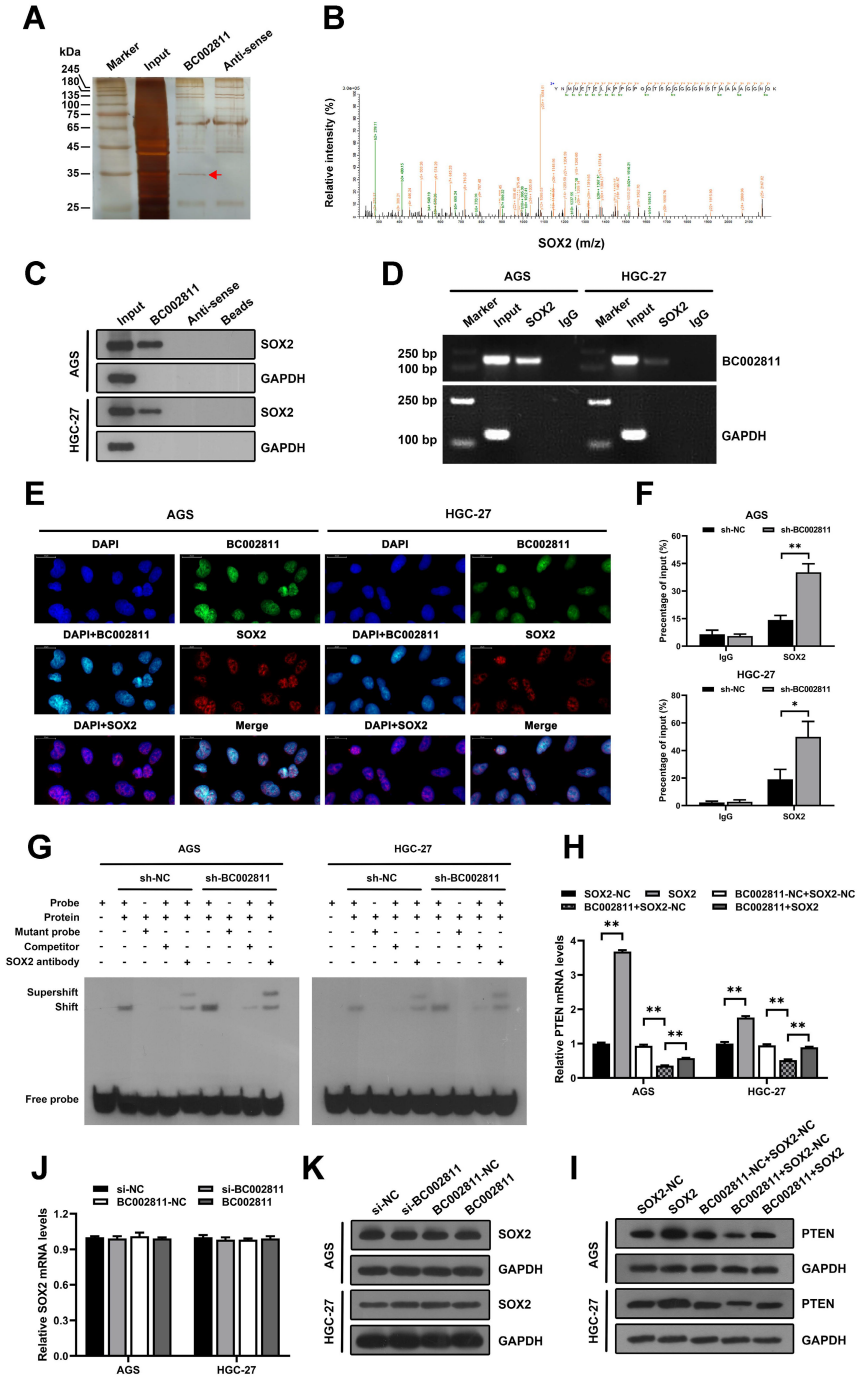
** BC002811 inhibits PTEN transcription by acting as a molecular decoy for the transcription factor SOX2. (A)** RNA pull-down assays were performed with HGC-27 cell lysates using full-length BC002811 and its anti-sense RNA, followed by SDS-PAGE and silver staining. A protein band specific to BC002811 (red arrow) was submitted for mass spectrometric identification. **(B)** MS/MS profiles of target bands (corresponding peptide sequences of SOX2) pulled down by BC002811. **(C)** BC002811 bound to SOX2 in AGS and HGC-27 cell lysates as shown by western blot analysis after RNA pull-down. **(D)** RIP assays of BC002811 binding to SOX2 in AGS and HGC-27 cell extracts. **(E)** RNA FISH analysis of BC002811 (green) and immunofluorescence detection of SOX2 (red) in AGS and HGC-27 cells. Confocal microscopy images showing colocalization of BC002811 and SOX2 in AGS and HGC-27 cells. Scale bar, 20 μm. **(F)** ChIP assays were performed in AGS and HGC-27 cells using SOX2 antibodies or IgG. The *PTEN* promoter fragment was detected by qPCR using specific primers. **(G)** EMSA showed SOX2 occupancy on the *PTEN* promoter regions, and that knockdown of BC002811 increased SOX2's occupancy. qPCR **(H)** and western blot **(I)** analyses were performed to determine the PTEN expression after upregulation of SOX2 in AGS and HGC-27 cells overexpressing BC002811. The mRNA and protein levels of SOX2 were detected in AGS and HGC-27 cells by qPCR **(J)** and western blot **(K)** after knockdown or overexpression of BC002811. Data expressed as mean±SD. *P < 0.05, **P <0.01.

Recent studies have reported that lncRNAs affect GC metastasis through transcriptional and epigenetic regulation of oncogenes or tumor suppressors as a result of their interaction with proteins 18-20. To explore whether BC002811 affects GC cell metastasis in such a manner, we sought to identify proteins associated with BC002811 in GC cells using RNA pull-down assays. After SDS-PAGE and silver staining, the protein bands specific to BC002811 pull-down were subjected to mass spectrometry (Fig. 6A). The transcription factor SOX2 was the main protein identified by mass spectrometry analysis (Fig. 6B and Table S4), and this was confirmed by western blotting (Fig. 6C). To further confirm the interaction between BC002811 and SOX2, we performed RNA immunoprecipitation (RIP) using an antibody against SOX2. We observed BC002811 enrichment in SOX2 antibody RIPs relative to the IgG control antibody (Fig. 6D). In addition, BC002811 fluorescent *in situ* hybridization (FISH) followed by immunofluorescence of SOX2 demonstrated co-localization of BC002811 with SOX2 in the nucleus of AGS and HGC-27 cells (Fig. 6E), implying a potential role of BC002811 in transcriptional regulation of genes via interaction with SOX2. *PTEN* is a well-known tumor suppressor gene and has been shown to be a direct target of transcriptional regulation by SOX2 in GC 16. To determine whether BC002811 affects the binding of SOX2 to the *PTEN* promoter, ChIP and EMSA analyses were carried out with GC cells. The ChIP assays indicated that BC002811 knockdown enhanced SOX2 binding to the *PTEN* promoter (Fig. 6F). Similarly, downregulation of BC002811 expression also promoted SOX2 binding to the *PTEN* promoter, which was further validated by EMSA (Fig. 6G). These results indicate that BC002811 may serve as a molecular decoy of SOX2 by binding directly to SOX2, thereby preventing SOX2 from binding to the *PTEN* promoter. To verify whether BC002811 mediated inhibition of PTEN expression through SOX2, we performed rescue experiments. SOX2 overexpression was found to reverse the BC002811-mediated decrease in PTEN expression at the mRNA (Fig. 6H) and protein levels (Fig. 6I). However, neither knockdown nor upregulation of BC002811 changed SOX2 mRNA (Fig. 6J) or protein expression (Fig. 6K), thus ruling out a role of BC002811 in inhibition of PTEN transcription through downregulation of SOX2 expression. Together, these data demonstrate that BC002811 may inhibit PTEN transcription by interacting with SOX2.

### Combining BC002811 and PTEN expression provides elevated prognostic value

**Figure 7:**
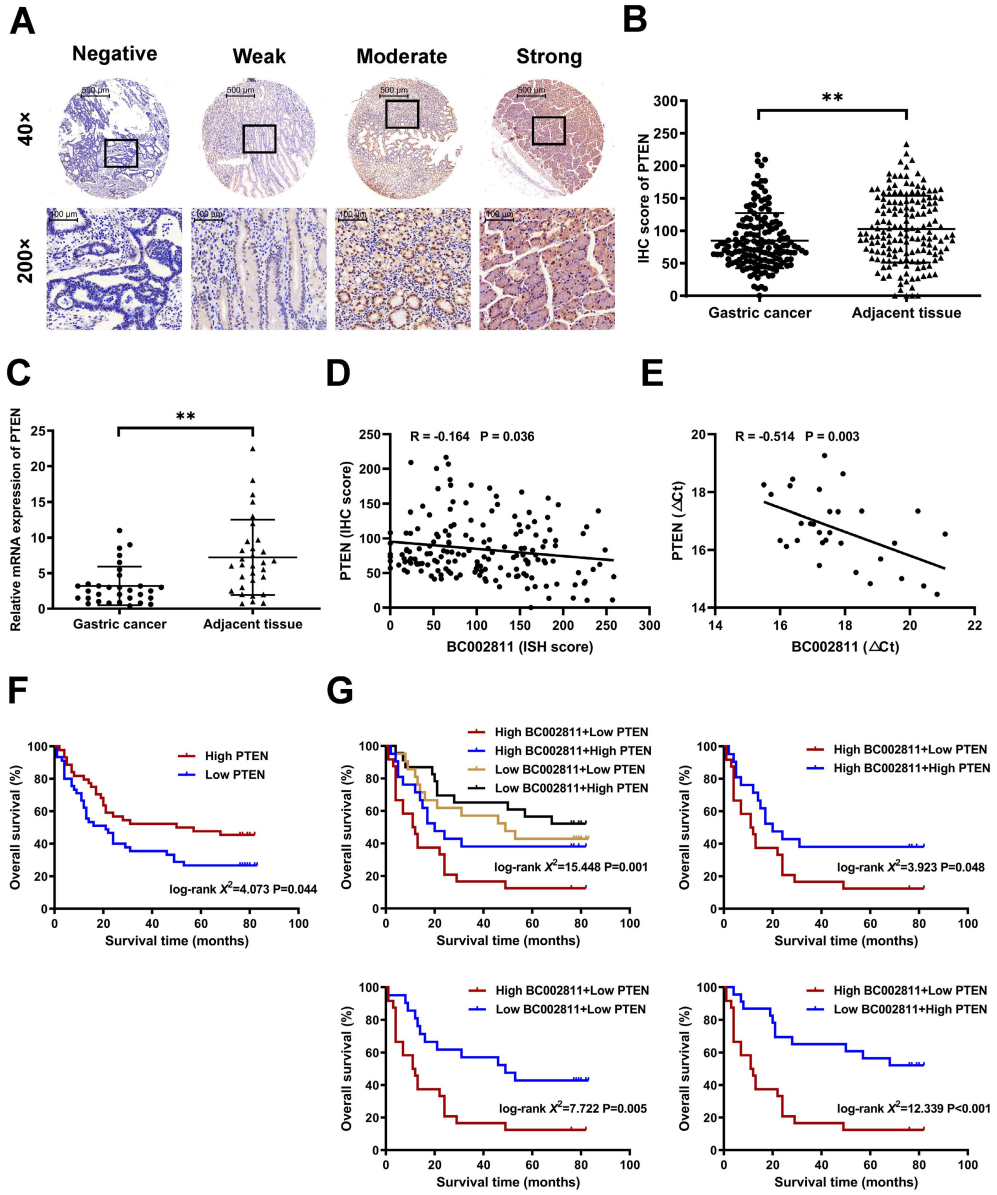
** Prognostic significance of BC002811 expression in combination with PTEN expression. (A)** Representative images of different intensities of PTEN immunohistochemistry (IHC) staining in tissue microarrays containing 163 cases of GC tissues and the adjacent non-tumorous gastric specimens (40× and 200× magnification). Scale bars, 500 μm (40× images) and 100 μm (200× images). **(B)** IHC scores for PTEN staining in GC tissue microarrays. **(C)** The mRNA expression of PTEN was analyzed by qPCR in 31 fresh-frozen GC tissue samples. **(D-E)** Analysis of the correlation between BC002811 expression and PTEN protein **(D)** and mRNA **(E)** levels in GC tissues. **(F)** Kaplan-Meier survival analysis of GC patient overall survival according to PTEN expression. All GC patients were segregated into “High PTEN” or “Low PTEN” expression groups using the median expression level of PTEN as the cut-off point. **(G)** When grouped by BC002811 expression as well as PTEN expression, Kaplan-Meier survival analysis indicated that patients with high BC002811 expression combined with low PTEN expression had a poor-prognostic signature. Data expressed as mean±SD. **P < 0.01.

We measured PTEN expression by immunohistochemistry and qPCR in paired samples from GC tissue microarrays and surgically resected GC samples to assess the pathological correlation between BC002811 and PTEN levels in clinical samples. We found that PTEN protein levels in GC tissues were lower than those in their paired non-tumorous gastric specimens (Fig. 7A-B and Fig. S1C-D). Similarly, the mRNA levels of PTEN were also lower in GC samples relative to the matched non-tumorous regions (Fig. 7C), which is consistent with a previous report 16. Correlation analysis revealed that BC002811 expression was inversely correlated with PTEN protein (Fig. 7D) and mRNA levels (Fig. 7E), in keeping with the action of BC002811 in the regulation of PTEN expression. The outcomes with respect to the impacts of PTEN expression on overall survival (OS) of GC patients revealed that GC patients with low PTEN expression had slightly worse prognoses (Fig. 7F). To achieve better clinical GC prognostication, GC patients were classified into four subgroups based on the expression levels of BC002811 and PTEN. Kaplan-Meier analysis revealed significantly distinct survival patterns between these subgroups, whereby the patients with upregulated BC002811 expression and downregulated PTEN expression had decreased OS than those in the other subgroups (Fig. 7G). As the High-BC002811/Low-PTEN subgroup was correlated with markedly decreased OS compared to the Low-BC002811/High-PTEN subgroup (Fig. 7G), this indicates a negative correlation between BC002811 and PTEN in GC prognosis. These results also highlight the clinical value of a suitable prognostic profile in GC, which can be achieved by a combined signature of BC002811 and PTEN expression.

## Discussion

A majority of patients with GC die from metastatic disease, but the detailed molecular mechanisms underlying GC metastasis are still only partially understood. In this study, we identified a lncRNA, BC002811, that was upregulated in GC tissues and cell lines and that correlated with reduced patient OS. Using overexpression and knockdown experiments, BC002811 was found to be essential for GC cell growth and metastasis *in vitro* and *in vivo*. Our results strongly suggest that BC002811 is an oncogenic lncRNA in GC.

The role of BC002811 in GC metastasis appears to involve regulation of MMP2, MMP9, and PTEN. MMP2 and MMP9 (two gelatinases that are members of the matrix metalloproteinase family) can facilitate malignant cell progression and promote tumor growth, invasion, and metastasis owing to their ability to degrade type IV collagen in the extracellular matrix and basement membrane 21,22. MMP2 and MMP9 are molecular markers of tumor invasion and metastasis, including GC, and inhibition of their expression reduces the invasion and migration capabilities of GC cells 22-24. MMP2 and MMP9 have also been reported to participate in the development and progression of GC, and they have been shown to have a predictive role in the aggressive behavior of GC 22,25. PTEN serves as a tumor suppressor gene, influencing cell proliferation, apoptosis, mobility, signal transduction, and other crucial cellular processes 26. Reduction of PTEN expression has been observed in multiple cases of GC and has been shown to be tightly correlated with the development, progression, and prognosis of this disease 15. PTEN knockdown also promotes GC cell metastasis *in vitro* and *in vivo* 16,27. Here, we found that BC002811 upregulation inhibited PTEN and promoted MMP2 and MMP9 expression, while silencing of BC002811 resulted in the opposite effects. Moreover, PTEN has been reported to inhibit cell invasion and migration by downregulation of the expression and enzymatic activity of MMP2 and MMP9 in multiple human cancers, including GC 27-30. These results suggest that BC002811 regulates GC metastasis through PTEN. Therefore, we deployed a BC002811 gain-of-function strategy in PTEN-overexpressing GC cells, and we found that BC002811 overexpression decreased the anti-invasive and anti-migratory effects of PTEN upregulation. In accordance with these results, decreased expression of MMP2 and MMP9, as well as the suppression of MMP2 and MMP9 enzyme activity caused by PTEN upregulation, were also reversed by BC002811 overexpression. These results indicated that BC002811 regulated MMP2 and MMP9 at least partly through PTEN, which is responsible for the metastasis-promoting effects of BC002811 in GC. However, the precise molecular mechanisms of PTEN regulation by BC002811 remain unclear.

Immunoprecipitation and mass spectrometric analyses of BC002811-interacting proteins led us to focus on the transcription factor SOX2. SOX2 is a critical regulator of embryogenesis and plays a major role in cell proliferation, differentiation, and cell fate determination 31,32. Recently, SOX2 has been implicated in the pathogenesis of GC, functioning as a tumor suppressor that reduces GC cell proliferation and metastasis while also promoting apoptosis 16,31,33,34. SOX2 loss has also been shown to enhance gastric tumorigenesis in an Apc-deficient mouse model 35. Moreover, PTEN has been identified as a direct target of transcriptional regulation by SOX2, and it is upregulated in response to SOX2-enforced expression, thereby leading to anti-metastatic effects in GC 16.

LncRNAs can play important roles in cancer initiation, progression, and metastasis through precise regulation of gene expression at the transcriptional and epigenetic levels by acting as scaffolds, guides, activators, or decoys for their interacting proteins, such as histone modifiers and transcription factors 36,37. In this study, we demonstrated an essential role for BC002811 in the transcriptional regulation of PTEN through interaction with the transcription factor SOX2 based on the following findings: (1) BC002811 downregulated PTEN expression at the transcriptional and translational level; (2) BC002811 could directly interact with SOX2; (3) knockdown of BC002811 increased the effects of SOX2 binding to the *PTEN* promoter; (4) BC002811 co-localized with SOX2 in the nucleus of GC cells, in keeping with previous reports that transcription and epigenetic regulation of genes can be influenced by nucleus-retained lncRNAs 38,39; (5) overexpression of SOX2 reversed the BC002811-mediated repression of PTEN expression, but BC002811 did not influence SOX2 expression. A similar regulatory mechanism has also been observed in another lncRNA, Lethe, which interacts with the transcription factor NF-κB subunit RelA to suppress RelA DNA binding activity and target gene activation 40.

Decreased expression of PTEN has been reported to be involved in worse patient outcomes in GC 15,16,41. Coincidentally, our findings indicate that both high BC002811 expression and low PTEN expression correlated with worse prognoses in GC patients. There is increasing evidence that a suitable combination of various biomarkers provides a higher degree of accuracy than a single biomarker in prognosis assessment 16,42. Here, we report that GC patients with elevated BC002811 levels and decreased PTEN expression displayed the lowest median survival time, indicating that increased value of combining BC002811 and PTEN in regard to prognostic accuracy for GC patients.

**Figure 8:**
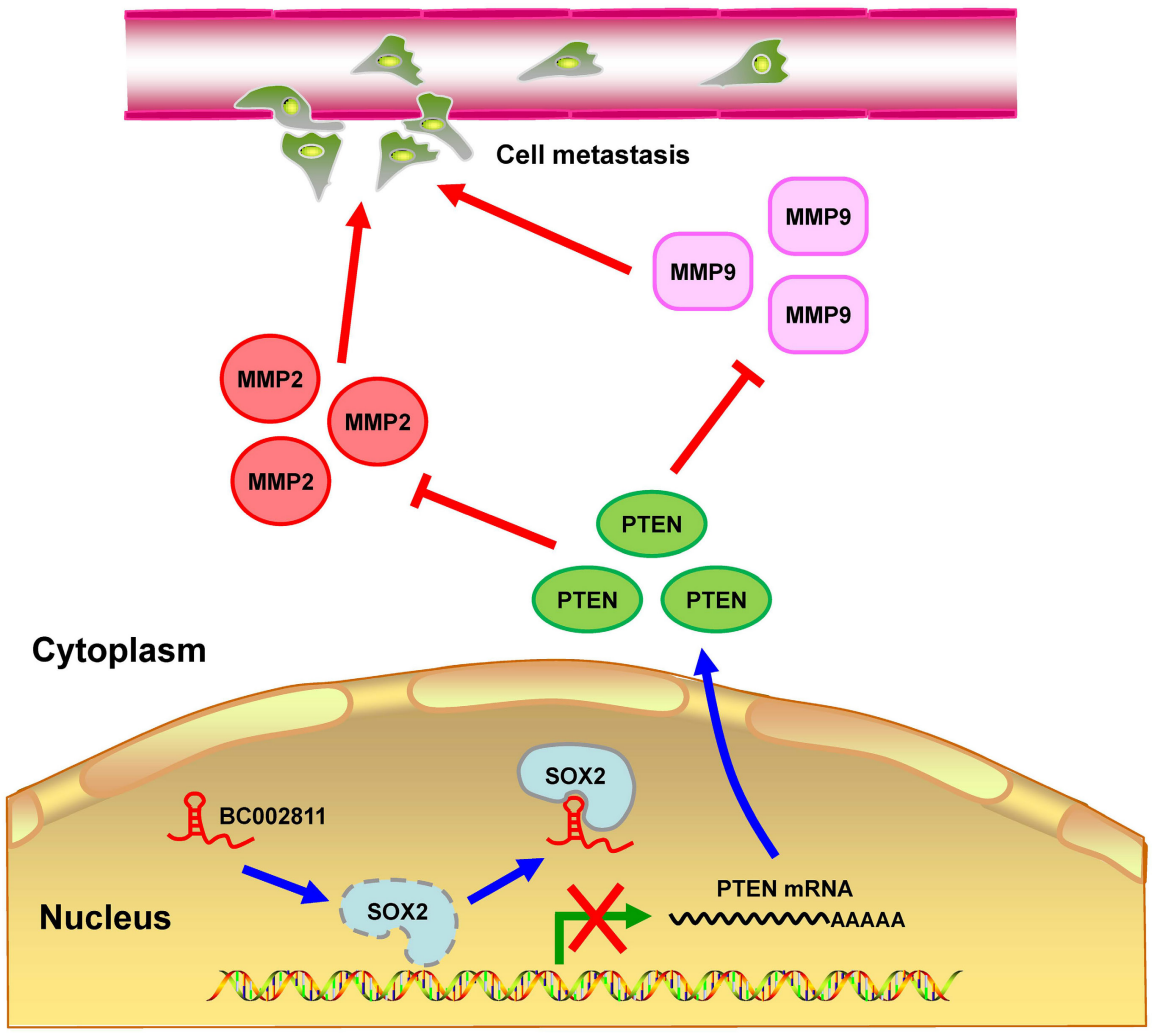
** Proposed model for BC002811 regulation of PTEN expression and GC metastasis.** BC002811 competitively SOX2 interaction with the *PTEN* promoter. This leads to suppression of PTEN transcription and promotion of MMP2 and MMP9 expression, thereby exerting its pro-metastatic role in GC.

In summary, we identified BC002811 as an oncogenic lncRNA involved in GC growth and metastasis. Our findings suggest that BC002811 binds to SOX2, thereby disrupting the interplay between SOX2 and the *PTEN* promoter, which consequently leads to suppression of PTEN transcription. The decrease in the level of PTEN protein can further stimulate MMP2 and MMP9 expression, which ultimately promotes GC cell metastasis (Fig. 8). Our study provides a potential strategy for the therapeutic intervention in GC metastasis by targeting oncogenic BC002811.

## Supplementary Material

Supplementary materials and methods, figure and tables.
