# Supplementary material for: Long non-coding RNA BC002811 Promotes Gastric Cancer Metastasis by Regulating SOX2 Binding to the PTEN Promoter
Source: Int J Biol Sci. 2023 Jan 16;19(3):967–80. doi: 10.7150/ijbs.76407 (PMC9909995; doi:10.7150/ijbs.76407)
Supplement: Supplementary file 1 — Supplementary materials and methods, figure and tables. [file ijbsv19p0967s1.pdf]

Supplementary Material

Supplementary Materials and Methods

Human GC samples

Fresh samples of human GC and corresponding adjacent non-tumorous tissues were obtained from 31 patients who underwent surgical resection in the Department of Gastrointestinal Surgery at Shenzhen People's Hospital (Shenzhen, China) between May 2013 and May 2015. None of the patients received chemotherapy or radiotherapy before the surgery. This investigation was sanctioned by the ethics committee of the Shenzhen People's Hospital. Written informed consent was obtained from all participants. All tissue samples were immediately frozen and stored in liquid nitrogen until needed for further testing.

Cell culture

GES-1 and HGC-27 cells were obtained from Shanghai Cell Bank of the Chinese Academy of Sciences (Shanghai, China). AGS, Hs746T and NCI-N87 cells were obtained from the American Type Culture Collection (Manassas, VA, USA). GES-1 cells were cultured in DMEM medium (Gibco, Carlsbad, CA, USA); AGS, HGC-27, Hs746T and NCI-N87 cells were maintained in RPMI 1640 medium (Gibco) supplemented with 10% fetal bovine serum (HyClone, Logan, UT) at 37 ℃ in 5% CO_2.

RNA extraction and quantitative real-time PCR (qPCR)

Total RNA was isolated with Trizol reagent (Invitrogen, Carlsbad, CA, USA) and reverse transcribed into cDNA using an M-MLV reverse transcriptase kit (Promega, Mannheim, Germany) according to the respective manufacturer's instructions. QPCR was performed on ABI Prism 7500 Real-Time PCR System (Applied Biosystems, Foster, CA, USA) using Platinum SYBR Green qPCR SuperMix-UDG Kits (Invitrogen) following the protocols provided by the manufacturer. Cycling conditions

were 95 ℃ for 10min (initial denature), followed by 40 cycles at 95 ℃ for 10 sec and 60 ℃ for 1 min. All primers sequences used in this study were shown in Table S1. Relative expression was calculated by using the comparative $2^{-\Delta\Delta Ct}$ method.

SiRNAs, plasmids transfection and establishment of stable cell lines

BC002811 siRNA and scrambled siRNA (Table S1) were designed and synthesized by Sigma-Aldrich (St. Louis, MO, USA). BC002811 expression vector, BC002811 shRNA vector, PTEN expression vector and SOX2 expression vector were purchased from Land Unicomed (Guangzhou, China). Transfection of plasmid vectors was conducted using Lipofectamine 2000 reagent (Invitrogen), while siRNAs were transfected using RNAiMax (Invitrogen) according to the manufacturer's instructions. For stable knockdown of BC002811, GC cells were infected with lentivirus-mediated shRNA targeting BC002811. To obtain cell lines stably expressing BC002811, cells were infected with lentivirus expressing BC002811. All infected GC cells were selected with 4μg/ml puromycin for 2 weeks.

Cell proliferation assay

For cell viability assay, GC cells were seeded onto 96-well plates (1×10^4 cells per well) after transfection, and cell viability was monitored every 24 h at a wavelength of 490 nm using a CellTiter96AQ Cell Proliferation Assay kit (MTS) (Promega) following the manufacturer’s standard workflow. For colony formation assay, 200 transfected cells were plated into each well of 6-well plates and cultured in complete medium. Approximately 7 days later, the cells were fixed with 4% paraformaldehyde and stained with 0.1% crystal violet (Beyotime, Shanghai, China). Visible colonies were then counted under a microscope and the colony formation efficiency (CFE) was calculated as previously described by Lawrenson et al (Lawrenson K, Grun B, Benjamin E, Jacobs IJ, Dafou D, Gayther SA. Senescent fibroblasts promote neoplastic transformation of partially transformed ovarian epithelial cells in a three-dimensional model of early stage ovarian cancer. Neoplasia. 2010 Apr;12(4):317-25.).

Flow cytometric analysis of apoptosis

Cell apoptosis assay was performed on FACSCalibur flow cytometer (BD Biosciences, Sparks, MD, USA) using Annexin V-PE/7-AAD Apoptosis Detection Kit (Solarbio, Beijing, China) according to the manufacturer's directions. Briefly, GC cells were harvested and resuspended in binding buffer at a concentration of 1×10^6 cells/ml. This suspension (500 μl) was incubated with 5 μl of Annexin V-PE and 10 μl of 7-AAD in the dark at room temperature for 15 min, followed immediately by flow cytometric analysis.

Cell migration and invasion assays

For migration assay, 1×10^5 GC cells were suspended in 100 μl serum-free medium and seeded on the membrane of the upper chamber of 24-well Transwell plates (Corning, NY, USA) at 24 h post-transfection. For invasion assay, GC cells (1×10^5) in serum-free media were placed into the upper chamber of Transwell plates pre-coated with Matrigel (BD Biosciences, San Jose, CA, USA). Subsequently, 600 μl of complete medium was added to the lower chamber. After 24 h, the cells on the upper membrane were gently scraped away using cotton wool, whereas the cells on the lower membrane were fixed with 4% paraformaldehyde and stained with 0.1% crystal violet (Beyotime) followed by microscopic observation and cell count.

Western blot analysis

Cells were lysed in western and IP lysis buffer (Beyotime) supplemented with phenylmethylsulfonyl fluoride (Roche, CA, USA). The protein concentration was measured using the BCA Protein Assay Kit (KeyGEN Biotech, Nanjing, China). Equal amounts of protein were separated by SDS-PAGE and transferred onto a polyvinylidene fluoride (PVDF) membrane (Millipore, Billerica, MA, USA). The membranes were blocked in Western blocking buffer (Beyotime) for 1 h at room temperature, followed by incubation with primary antibodies overnight at 4 ℃. After three 10-min washes in Tris-buffered saline solution with Tween (TBST), the

membranes were incubated with horseradish peroxidase (HRP)-conjugated secondary antibodies for 1 h at room temperature. The protein bands were visualized with an enhanced chemiluminescence reagent (Millipore, Billerica, MA, USA).

Gelatin zymography

The enzyme activity of secreted MMP2 and MMP9 was measured by gelatin zymography as previously described (Xu TP, Huang MD, Xia R, Liu XX, Sun M, Yin L, Chen WM, Han L, Zhang EB, Kong R, De W, Shu YQ. Decreased expression of the long non-coding RNA FENDRR is associated with poor prognosis in gastric cancer and FENDRR regulates gastric cancer cell metastasis by affecting fibronectin1 expression. J Hematol Oncol. 2014 Aug 29;7:63. doi: 10.1186/s13045-014-0063-7.), with several modifications. Briefly, a total of 40 μg of protein from the conditioned medium of GC cells was loaded on 10% SDS-PAGE gels containing 0.1% gelatin (Sigma-Aldrich) under non-reducing conditions. After electrophoresis at 80 V for 2.5 h at 4 ℃, the gel was washed twice in 2.5% Triton-X 100 and incubated in developing buffer (50 mM Tris–HCl, pH 7.8, 5 mM $CaCl_2$, 0.2M NaCl and 0.02% Brij 35) for 42 h at 37 ℃. Subsequently, the gel was stained with 0.5% Coomassie blue R250 (Sigma-Aldrich) and destained in 30% methanol and 10% acetic acid. Gelatinolytic activity was identified as transparent bands on the gel.

Statistical analysis

All the statistical analyses were carried out using SPSS 16.0. Statistical data were presented as the mean ± standard deviation (SD) from at least three replicates. Differences between experimental groups were evaluated by Wilcoxon signed-rank test, one-way analysis of variance or Student's t-test, respectively. Correlations between BC002811 expression and clinicopathological parameters were assessed using Pearson's chi-square test. Kaplan–Meier analysis and log-rank test were utilized to analyze the relationship between BC002811 expression and overall survival (OS). Pearson correlation analyses were carried out to investigate the correlation between BC002811 and PTEN expressions. Differences were considered to be statistically

significant when $P < 0.05$.

Supplementary Figures

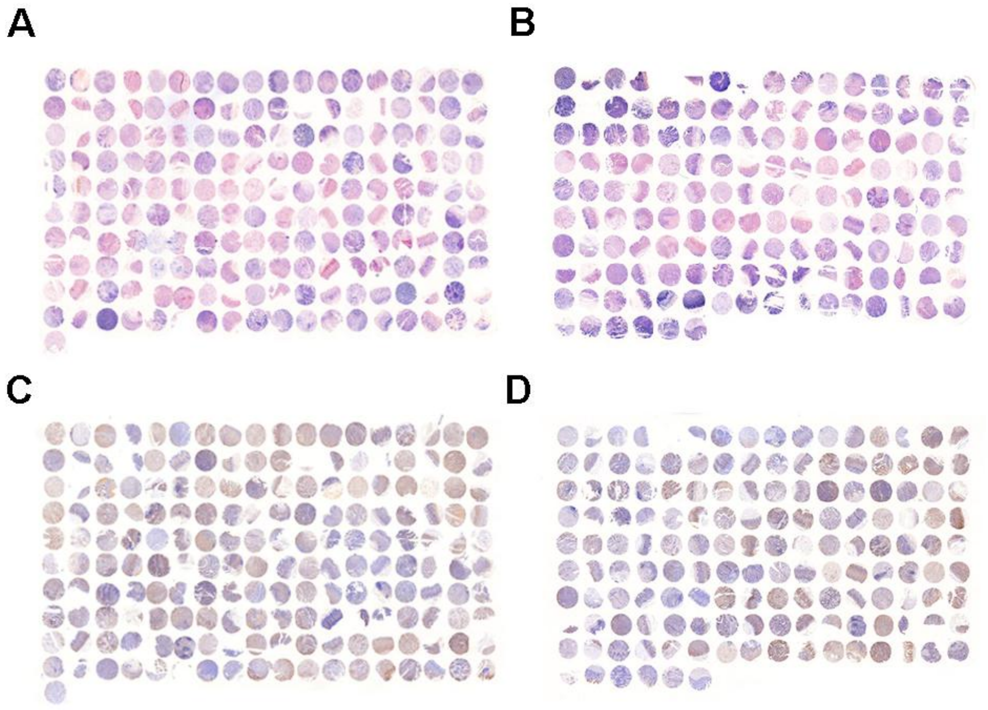

Figure S1. Overview of the tissue microarray results in GC. In situ hybridization assays of BC002811 in 90 cases (A) and 75 cases (B) of gastric cancer tissue microarrays. Immunohistochemistry assays of PTEN in 90 cases (C) and 75 cases (D) of gastric cancer tissue microarrays.

Supplementary Tables

Table S1. Oligonucleotide sequences and primers for this study.

	Name	Sequence (5'-3')
BC002811 siRNA	Sense	CUCCUGACCUCAGUUCAUCdTdT
	Anti-sense	GAUGAACUGAGGUCAGGAGdTdT
Scrambled siRNA	Sense	UUCUCCGAACGUGUCACGUTT
	Anti-sense	ACGUGACACGUUCGGAGAATT
qPCR or RT-PCR primer		
BC002811	Sense	CTTCCAAAGTGCTGGGATTA
	Anti-sense	TCCCAGTTTTATAGTTTTGT
U6 snRNA	Sense	CTCGCTTCGGCAGCACA
	Anti-sense	AACGCTTCACGAATTTGCGT
MMP2	Sense	CTGGAGATACAATGAGGTGAAG
	Anti-sense	TCTGAGGGTTGGTGGGATTG
MMP9	Sense	GAACCAATCTCACCGACAGG
	Anti-sense	GCCACCCGAGTGTAACCATA
PTEN	Sense	TTGTGGTCTGCCAGCTAAA
	Anti-sense	CGCTCTATACTGCAAATGCT
18S rRNA	Sense	CCTGGATACCGCAGCTAGGA
	Anti-sense	GCGGCGCAATACGAATGCCCC
GAPDH	Sense	GCTCATTTGCAGGGGGGAG
	Anti-sense	GTTGGTGGTGCAGGAGGCA
ISH probe		
	BC002811	TAAGGTTCTGAGGATGGATAT
RNA-FISH probe		
	BC002811	CY3-TAAGGTTCTGAGGATGGATAT-CY3
ChIP-qPCR primer		
PTEN promoter	Sense	GATACACGCTGGCGACACAA
	Anti-sense	TCGCTCTTTCCTTTTGCACC

Table S2. Correlation between BC002811 expression and clinicopathological parameters of gastric cancer.

Clinical parameter	BC002811		Chi-square	P value
	High (n)	Low (n)		
All cases	82	81		
Age			2.713	0.1
<60	31	41		
≥60	51	40		
Gender			1.457	0.227
male	57	49		
female	25	32		
Tumor size			9.630	0.002**
<5 cm	23	42		
≥5 cm	59	39		
Invasion depth			0.715	0.398
T1/T2	13	17		
T3/T4	69	64		
TNM stage			6.725	0.010*
I/II	28	44		
III/IV	54	37		
Lymph node metastasis			5.946	0.015*
N0/N1	28	43		
N2/N3	54	38		

Clinicopathological parameters were evaluated with Pearson's chi-square test. *$P < 0.05$, **$P < 0.01$.

Table S3. Differentially metastasis-related mRNA expression profiling by human tumor metastasis qPCR array.

Gene symbol	RefSeq	Ct value			Fold Up- or Down-Regulation	
		BC002811	NC	sh-BC002811	BC002811 vs. NC	sh-BC002811 vs. NC
APC	NM_000038	24.400797	24.0037	24.67994	-1.529478259	-1.599965504
BRMS1	NM_015399	21.528267	21.75821	21.598715	1.009751578	1.11550285
CCL7	NM_006273	31.278658	32.62274	30.376188	2.185766667	4.739553653
CD44	NM_000610	20.818434	20.288452	20.673094	-1.677048718	-1.307166395
CD82	NM_002231	23.395935	24.445812	24.4407	1.782539937	1.002297785
CDH1	NM_004360	32.069283	31.811382	34.261272	-1.38880457	-5.470568632
CDH11	NM_001797	31.045656	30.951212	30.913454	-1.24003995	1.025236822
CDH6	NM_004932	25.156984	24.3489	25.331575	-2.033586841	-1.978594264
CDKN2A	NM_000077	20.205362	20.171194	20.32553	-1.189298286	-1.114299306
CHD4	NM_001273	18.436754	18.564262	18.570107	-1.063215911	-1.005313733
COL4A2	NM_001846	31.341059	34.13883	32.661736	5.986979789	2.780401445
CST7	NM_003650	29.699316	29.493563	28.960459	-1.339500946	1.4452341
CTBP1	NM_001328	27.114796	26.915947	26.814629	-1.333106094	1.071414852
CTNNA1	NM_001903	19.51758	19.493656	19.654633	-1.180883465	-1.119440462
CTSK	NM_000396	20.78995	20.50296	20.902657	-1.417091191	-1.32087853
CTSL	NM_001912	21.643251	21.753565	21.872078	-1.075963099	-1.086971263
CXCL12	NM_000609	35.230354	33.339993	33.76481	-3.670431302	-1.344078872
CXCR2	NM_001557	25.015753	24.826986	25.360443	-1.323822411	-1.449201094
CXCR4	NM_003467	26.21628	26.679264	26.687954	1.186770853	-1.007298171
DENR	NM_003677	20.615705	20.613712	20.881521	-1.163068123	-1.205481742
EPHB2	NM_004442	24.628904	24.595375	24.748638	-1.188771637	-1.113470858
ETV4	NM_001986	30.79483	31.124884	31.78728	1.082308213	-1.584685776
EWSR1	NM_005243	21.269636	21.194498	21.341368	-1.223556396	-1.108547662
FAT1	NM_005245	20.389057	20.104902	20.37925	-1.414309239	-1.210957976
FGFR4	NM_002011	28.76574	29.224815	29.33431	1.183559635	-1.080198013
FLT4	NM_002020	30.521866	30.92661	30.65032	1.139816356	1.209565755
FN1	NM_002026	22.3933	22.415754	23.21283	-1.14352555	-1.739746132
FXYD5	NM_014164	20.431787	20.382574	20.402924	-1.201765657	-1.015472239
GNRH1	NM_000825	27.469	26.812225	26.90704	-1.831108908	-1.069262298
HGF	NM_000601	29.406118	30.351194	30.385931	1.657643077	-1.025649503
HPSE	NM_006665	25.229996	25.329588	25.475443	-1.083989363	-1.107768024
HRAS	NM_005343	21.710443	22.202272	20.97667	1.210737745	2.335612925
HTATIP2	NM_006410	32.167651	31.632256	32.26264862	-1.683352834	-1.549919643
IGF1	NM_000618	25.67241	24.510439	25.002403	-2.598922476	-1.408114646
IL18	NM_001562	31.207802	31.900448	31.744505	1.391560275	1.112759795
IL1B	NM_000576	33.177063	31.834532	32.553802	-2.945420472	-1.648405063
ITGA7	NM_002206	26.120579	26.388163	26.157919	1.036443448	1.171570036

ITGB3	NM_000212	26.646856	26.30055	26.425827	-1.476568821	-1.092079437
KISS1	NM_002256	30.253904	30.153698	31.82514	-1.245002466	-3.189306609
KISS1R	NM_032551	32.504566	31.996613	32.832355	-1.651635806	-1.787003921
KRAS	NM_004985	21.982685	21.797384	22.186947	-1.320645814	-1.311632723
MCAM	NM_006500	22.83348	22.984993	24.726795	-1.04567143	-3.348703864
MDM2	NM_002392	24.871664	25.366825	25.569256	1.213537256	-1.152072733
MET	NM_000245	25.884682	26.434135	26.325628	1.260075694	1.076767074
METAP2	NM_006838	21.641228	21.548365	22.012276	-1.238681777	-1.380998545
MGAT5	NM_002410	26.048311	26.265476	25.899998	1.000847662	1.286701318
MMP10	NM_002425	30.356787	30.389212	30.555023	-1.135649469	-1.123197633
MMP11	NM_005940	29.56676	29.30755	30.104929	-1.390065245	-1.740111558
MMP13	NM_002427	UND	33.620632	34.553673	N/A	-1.911680997
MMP2	NM_004530	19.772665	21.158667	22.292572	2.250209615	-2.197260316
MMP3	NM_002422	UND	34.17379	UND	N/A	N/A
MMP7	NM_002423	38.644024	39.93651	UND	-1.16146252	N/A
MMP9	NM_004994	28.648964	29.884037	31.50213	2.026696978	-3.073524105
MTA1	NM_004689	22.078983	21.922129	22.015024	-1.294860429	-1.067840224
MTSS1	NM_014751	20.833866	20.29662	20.22868	-1.685513988	1.046911289
MYC	NM_002467	18.307337	17.989698	18.151814	-1.447518313	-1.120324603
MYCL	NM_005376	33.5963	34.099674	33.142902	1.220465404	1.938540935
NF2	NM_000268	24.987833	26.724075	26.74648	2.86850573	-1.016919726
NME1	NM_000269	17.784332	17.707243	17.998276	-1.225212168	-1.225044197
NME4	NM_005009	25.994476	25.650154	25.782202	-1.474539633	-1.097216939
NR4A3	NM_006981	30.522398	29.604443	30.4792	-2.194508107	-1.83598949
PLAUR	NM_002659	25.643324	25.433584	25.659914	-1.343207881	-1.171316372
PNN	NM_002687	19.84218	19.559113	20.276146	-1.413243048	-1.645851076
PTEN	NM_000314	26.048985	24.472029	23.152548	-3.465103872	2.492649785
RB1	NM_000321	21.942163	21.634604	22.126963	-1.437439863	-1.408500231
RORB	NM_006914	30.431238	29.125437	29.443459	-2.871378666	-1.248177231
RPSA	NM_002295	14.527325	14.513866	14.608362	-1.172348582	-1.069025895
SERPINE1	NM_000602	26.185825	26.123827	26.248543	-1.212462897	-1.091654858
SET	NM_003011	17.3959	17.319386	17.62882	-1.224723945	-1.240769218
SMAD2	NM_005901	20.439754	20.280413	20.460062	-1.297094508	-1.134022921
SMAD4	NM_005359	21.692614	21.518116	21.676344	-1.31079366	-1.117309442
SRC	NM_005417	26.2672	26.39584	26.731773	-1.062381994	-1.263769891
SSTR2	NM_001050	24.063967	24.063484	23.803364	-1.161851432	1.196084404
SYK	NM_003177	36.477234	35.868095	35.229908	-1.16146252	-1.001248999
TCF20	NM_005650	23.014574	23.001215	23.190908	-1.172267324	-1.141945501
TGFB1	NM_000660	20.627523	20.722246	20.540184	-1.087653936	1.133089013
TIMP2	NM_003255	18.30532	18.386501	18.230337	-1.097911373	1.112930267
TIMP3	NM_000362	21.487204	21.467165	21.233364	-1.177707769	1.174462134
TIMP4	NM_003256	34.246433	36.016335	34.927757	1.451580317	1.050038481
TNFSF10	NM_003810	27.055523	28.954768	28.591997	3.211622665	1.284289281
TP53	NM_000546	25.36515	25.49461	25.268162	-1.061778328	1.168491468

TRPM1	NM_002420	UND	UND	UND	N/A	N/A
TSHR	NM_000369	30.496893	29.832523	30.229504	-1.840774113	-1.318394199
VEGFA	NM_003376	19.735508	19.670408	19.42233	-1.21507267	1.186142383
ACTB	NM_001101	13.786638	14.344679	13.845302	1.267598977	1.411839607
B2M	NM_004048	17.790333	17.898544	17.962105	-1.077532661	-1.046347311
GAPDH	NM_002046	13.288732	13.603623	13.804002	1.070992517	-1.150435261
HPRT1	NM_000194	20.33128	20.566963	20.677183	1.013777043	-1.080740983
RPLP0	NM_001002	13.879562	13.742449	13.858662	-1.277262983	-1.085239753
HGDC	SA_00105	UND	UND	UND		
RTC	SA_00104	UND	UND	UND		
RTC	SA_00104	UND	UND	UND		
RTC	SA_00104	UND	UND	UND		
PPC	SA_00103	18.909622	19.241392	18.934938		
PPC	SA_00103	18.753391	19.025349	18.942236		
PPC	SA_00103	18.95349	18.977577	18.77811		

HGDC represents the human genomic DNA contamination.

RTC is used as reverse transcription control.

PPC is used as positive PCR control.

N/A, not available.

UND, Undetermined.

Table S4. Mass spectrometry analysis of a specific protein band pulled down by lncRNA BC002811.

Protein ID	Protein Name	Gene Symbol	UnusedScore	Peptides*
P48431	Transcription factor SOX-2	SOX2	280.37	559
P08670	Vimentin	VIM	119.88	144
P04264	Keratin, type II cytoskeletal 1	KRT1	61.30	55
P13645	Keratin, type I cytoskeletal 10	KRT10	39.32	34
P35527	Keratin, type I cytoskeletal 9	KRT9	37.73	38
P02533	Keratin, type I cytoskeletal 14	KRT14	30.16	25
P35908	Keratin, type II cytoskeletal 2 epidermal	KRT2	21.18	22
P48668	Keratin, type II cytoskeletal 6C	KRT6C	13.74	22
P16403	Histone H1.2	HIST1H1C	13.60	13
P07477	Trypsin-1	PRSS1	13.06	18
P07910	Heterogeneous nuclear ribonucleoproteins C1/C2	HNRNPC	7.87	8
P08779	Keratin, type I cytoskeletal 16	KRT16	7.47	20
P38646	Stress-70 protein, mitochondrial	HSPA9	7.09	3
P63261	Actin, cytoplasmic 2	ACTG1	6.85	5
P05787	Keratin, type II cytoskeletal 8	KRT8	6.33	10
Q86YZ3	Hornerin	HRNR	4.70	3
P13647	Keratin, type II cytoskeletal 5	KRT5	4.40	17
P06576	ATP synthase subunit beta, mitochondrial	ATP5F1B	4.26	2
P05783	Keratin, type I cytoskeletal 18	KRT18	4.13	6
O14979	Heterogeneous nuclear ribonucleoprotein D-like	HNRNPDL	3.71	3
P02768	Serum albumin	ALB	3.66	3
P22626	Heterogeneous nuclear ribonucleoproteins A2/B1	HNRNPA2B1	3.41	5
P30050	60S ribosomal protein L12	RPL12	3.16	2
Q04695	Keratin, type I cytoskeletal 17	KRT17	2.91	14
P07197	Neurofilament medium polypeptide	NEFM	2.74	4

* Number of peptides with >95% confidence score used for quantitation.
